# Enhanced Theta-Band Coherence Between Midfrontal and Posterior Parietal Areas Reflects Post-feedback Adjustments in the State of Outcome Uncertainty

**DOI:** 10.3389/fnint.2019.00014

**Published:** 2019-04-24

**Authors:** Yulia M. Nurislamova, Nikita A. Novikov, Natalia A. Zhozhikashvili, Boris V. Chernyshev

**Affiliations:** ^1^Laboratory of Cognitive Psychophysiology, National Research University Higher School of Economics, Moscow, Russia; ^2^Centre for Cognition & Decision Making, National Research University Higher School of Economics, Moscow, Russia; ^3^Center for Neurocognitive Research (MEG-Center), Moscow State University of Psychology and Education, Moscow, Russia; ^4^Department of Higher Nervous Activity, Lomonosov Moscow State University, Moscow, Russia

**Keywords:** cognitive control, decision making, error detection, theta oscillations, alpha oscillations, posterior parietal cortex, functional connectivity

## Abstract

Medial frontal cortex is currently viewed as the main hub of the performance monitoring system; upon detection of an error committed, it establishes functional connections with brain regions involved in task performance, thus leading to neural adjustments in them. Previous research has identified targets of such adjustments in the dorsolateral prefrontal cortex, posterior cortical regions, motor cortical areas, and subthalamic nucleus. Yet most of such studies involved visual tasks with relatively moderate cognitive load and strong dependence on motor inhibition – thus highlighting sensory, executive and motor effects while underestimating sensorimotor transformation and related aspects of decision making. Currently there is ample evidence that posterior parietal cortical areas are involved in task-specific neural processes of decision making (including evidence accumulation, sensorimotor transformation, attention, etc.) – yet, to our knowledge, no EEG studies have demonstrated post-error increase in functional connectivity in the theta-band between midfrontal and posterior parietal areas during performance on non-visual tasks. In the present study, we recorded EEG while subjects were performing an auditory version of the cognitively demanding attentional condensation task; this task involves rather non-straightforward stimulus-to-response mapping rules, thus, creating increased load on sensorimotor transformation. We observed strong pre-response alpha-band suppression in the left parietal area, which presumably reflected involvement of the posterior parietal cortex in task-specific decision-making processes. Negative feedback was followed by increased midfrontal theta-band power and increased functional coupling in the theta band between midfrontal and left parietal regions. This could be interpreted as activation of the performance monitoring system and top–down influence of this system on the posterior parietal regions involved in decision making, respectively. This inter-site coupling related to negative feedback was stronger for subjects who tended to commit errors with slower response times. Generally, current findings support the idea that slower errors are related to the state of outcome uncertainty caused by failures of task-specific processes, associated with posterior parietal regions.

## Introduction

Execution of cognitively demanding tasks requires proper functioning of task-specific neural processes (such as evidence accumulation, sensorimotor transformation based on task rules) and non-specific processes (such as generalized motor inhibition) (Andersen and Cui, 2009; van Driel et al., 2012; Navarro-Cebrian et al., 2013); performance monitoring system is also essential for flexible adaptive behavior (Ullsperger, 2006). These processes are supported by sustained attention to relevant stimuli, retention of task rules, and inhibition of irrelevant motor programs (Ridderinkhof, 2002; Dudschig and Jentzsch, 2009; King et al., 2010; Danielmeier and Ullsperger, 2011; Cohen, 2014). These mechanisms viewed together can be referred to as cognitive control (Yeung, 2014).

Situations requiring an increase of cognitive control level produce activation in the structures that are located in medial frontal regions and usually referred to as performance monitoring system (Ullsperger, 2006; Yeung and Cohen, 2006; Yeung, 2014). One of such situations is error detection – either internal, or driven by an external negative feedback (Holroyd et al., 2004). The purpose of the monitoring system activation is to produce top–down influence on lower-level brain regions, leading to various neural adjustments aimed to improve subsequent task performance (Yeung, 2014). For example, post-error medial frontal activation could be correlated with activation of regions processing task-relevant information, inhibition of task-unrelated regions, and non-specific inhibition of motor structures (King et al., 2010). These neural adjustments could lead to corresponding behavioral adjustments such as post-error slowing, improvement of accuracy or reduction of interference (smaller effect of irrelevant features on the response time) (King et al., 2010; Danielmeier and Ullsperger, 2011; Danielmeier et al., 2011).

At the electrophysiological level, activation of the performance monitoring system (in situations that require increase of cognitive control) is associated with enhanced frontal midline theta (FMT) oscillatory power, with its main hub in the medial frontal cortex (Womelsdorf et al., 2010; Cavanagh and Frank, 2014). Increased FMT, observed immediately following errors, reflects internal error detection (van Driel et al., 2012; Cohen and van Gaal, 2013), while enhanced FMT power emerging after negative feedback is related to external error detection (Cohen et al., 2007; Luft et al., 2014).

In situations that activate the performance monitoring system, in addition to the FMT increase, theta-band phase coherence between midfrontal regions and various brain structures is usually increased (Cavanagh and Frank, 2014). According to communication-through-coherence hypothesis, phase coherence of oscillatory signals in two brain regions reflects functional coupling between these regions and increased communication between them (Varela et al., 2001; Pesaran et al., 2008; Fries, 2015). Consequently, the aforementioned theta-band coherence is believed to reflect top–down influence from the performance monitoring system onto systems implementing task-related functions that have to be adjusted (Koshel’kov and Machinskaia, 2010; De Pascalis et al., 2012; Cavanagh and Frank, 2014). For example, increased functional connectivity with midfrontal regions was reported for: (1) dorsolateral prefrontal cortex, which is believed to be crucial for cognitive control implementation (Cavanagh and Frank, 2014), (2) posterior parietal and occipital regions, involved in visual perception and attention (Cohen and van Gaal, 2013), (3) motor areas (Narayanan et al., 2013), and (4) subthalamic nucleus, which is involved in non-specific motor inhibition (Zavala et al., 2016). Top–down character of the theta-band interaction was confirmed in some cases by Granger causality analysis. For example, increase of directional theta-band connection from midfrontal to occipital region (but not in the opposite direction) was observed after errors in a visual task (Cohen and van Gaal, 2013).

Theta-band coherence between midfrontal region and its various targets increases in a relatively short time interval (several hundreds of milliseconds) after a triggering event, such as error commission, negative feedback or task-switching cue (Cavanagh et al., 2009; Cavanagh and Frank, 2014; Cooper et al., 2015). The top–down influence supported by this coherence is believed to initiate proactive neural adjustments in the target regions that last longer than the coherence itself. The exact mechanisms of producing such neural adjustments is not fully understood, but the effect itself is proved by the fact that the strength of coherence is correlated with the strength of behavioral adjustments (such as post-error slowing or improvent of accuracy) that occur at the next trial, i.e., long after the coherence increase terminates (Cavanagh et al., 2009; Cohen and van Gaal, 2013).

In the present work, we study post-error theta-band coherence in an auditory condensation task (Posner, 1964; Gottwald and Garner, 1975), which is a variant of perceptual decision-making task. In general, perceptual-driven decision making could be considered as continuous sensorimotor transformation that involves such stages as early sensory processing, sensory evidence integration, implementation of stimulus-to-response (S-R) mapping rules, motor preparation, and, ultimately, response commission. Successful completion of these stages is supported by such aspects of cognitive control as top–down attention, robust retention of task rules, and motor inhibition.

Different perceptual tasks rely on various aspects of cognitive control to a different extent. For example, in the widely used random dot pattern task, the crucial step is integration of sensory evidence, so the task critically depends on sensory attention (Rorie et al., 2010; Siegel et al., 2011). In contrast, typical tasks used in cognitive control studies, such as Stroop task, Eriksen flanker task, Simon task, or SART, depend mainly on successful motor inhibition (Ridderinkhof, 2002; Ridderinkhof et al., 2004; Dudschig and Jentzsch, 2009; van Driel et al., 2012), as they require suppression of automatic responses triggered by irrelevant stimulus features; attention is also important in these tasks as it allows to suppress these irrelevant features at the sensory level. However, all these classical tasks imply quite simple 1:1 S-R mapping rules, in which just one stimulus feature has to be mapped onto response, while all other features (if any) should be simply ignored. When sensory evidence about the relevant stimulus feature is accumulated and fast automatic response is inhibited, the subsequent response selection is usually straightforward.

The condensation task, which we used both in our previous studies (Novikov et al., 2015, 2017) and in the current study, is different from the aforementioned classical tasks as it implies a complex S-R mapping rule that involves feature conjunctions rather than single stimulus features(Posner, 1964; Gottwald and Garner, 1975). Our task involves two features with two levels each, and both features should be accounted for simultaneously in order to select a correct response from two possibilities; none of the features alone is enough to solve the task. Thus, the task performance critically depends on implementation of a complex 4:2 S-R mapping. In contrast, the condensation task does not imply any to-be-inhibited automatic responses, and the stimuli are relatively simple and unambiguous. Consequently, the critical aspect of cognitive control involved in the condensation task is stable retention of the S-R mapping rule, while motor inhibition and low-level sensory attention are of less importance than in more classical tasks. Another characteristic feature of our version of the condensation task is its auditory domain, while most of previous cognitive control studies used visual tasks. Thus, investigation of error-related processes occurring in the task we used could possibly improve current understanding of cognitive control mechanisms.

Sensorimotor transformation essentially involves both frontal premotor areas and posterior parietal areas, including the lateral intraparietal area (LIP), medial intraparietal area (MIP) and adjacent regions such as V6A and PEc, and it starts well before the actual response commission (Roitman and Shadlen, 2002; Andersen and Cui, 2009; Siegel et al., 2011; Breveglieri et al., 2014; Cui, 2014; Tosoni et al., 2014; de Lafuente et al., 2015; Bosco et al., 2016; Hadjidimitrakis et al., 2017; Piserchia et al., 2017). This process could be accompanied by alpha-band suppression (Van Der Werf et al., 2008, 2010), which is in agreement with the general idea that alpha suppression reflects activation of corresponding brain regions (Klimesch et al., 2007). In our previous study (Novikov et al., 2015), we observed strong pre-response alpha suppression in lateral posterior parietal regions, followed by lateral central regions. We interpret this observation as a hallmark of decision-making process, with reactivation and implementation of an appropriate S-R mapping rule being its crucial constituent part. Thus, we expect that error-related adjustments would occur in lateral posterior parietal regions involved in decision making, and that initiation of these adjustments would be reflected in theta-band synchronization between midfrontal and lateral posterior parietal regions.

In addition to errors related to failures of task-specific processes, another type of errors may be caused by excessive lowering of a non-specific motor threshold (van Driel et al., 2012). The latter condition leads to premature random responses (committed before the information processing can be completed), thus, some of such responses can be erroneous. In our previous study (Novikov et al., 2017), we demonstrated that under the condensation task this two types of errors could be distinguished by their response times (RTs). Slow errors may be associated with the state of high uncertainty, making the external feedback important for determining whether the response was erroneous. On the contrary, fast errors are supposedly related to the state of lowered motor threshold, under which the correct motor program continues to develop even after random response commission. This makes post-response internal error detection possible, thus, making feedback information less important.

Guided by these assumptions, we expected stronger synchronization between midfrontal and lateral parietal regions induced by the negative feedback for subjects who tended to commit slower errors. There are two rationales for this expectation: (1) after slow errors, which are associated with the state of uncertainty, feedback signal is the only source of information for the performance-monitoring system to initiate neural processes of post-error adjustments – while during fast errors internal error detection is possible before the feedback signal; (2) since slow errors – compared with fast errors – are more likely to be caused by failures of task-specific processes, adjustments of the systems that participate in implementation of these processes would be stronger after slow errors compared to fast ones.

In the present work, we aimed to investigate feedback-related interaction between the cognitive control monitoring system and the decision-making system. First, we demonstrated lateralized left central-parietal alpha suppression started well before response commission, i.e., during the decision making process. Next, we reproduced our previous results – an FMT increase after negative feedback, which is related to detection of the need for increased cognitive control. Third, we demonstrated increased theta-band coherence between midfrontal and lateral posterior parietal electrodes after negative feedback, which supposedly reflects top–down influence of the performance monitoring system on the decision-making system. Fourth, we demonstrated that the increase in theta-band coherence is more pronounced for those subjects who tend to commit slower erroneous responses.

## Materials and Methods

### Participants and Experimental Conditions

Fifty-one healthy right-handed volunteers participated in this study; their mean age was 23.2 ± 3.6 years (mean ± SD), 31 females, and 20 males.

### Stimuli

We used four pre-recorded auditory stimuli that differed in two independent features: timber (“cello” or “calliope”) and pitch (“low” 440 Hz, A4, or “high” 523.25 Hz, C5) (Table 1). The tones were synthesized using Microsoft “GS Wavetable SW Synth” integrated into Microsoft DirectX (Microsoft Corporation, Redmond, WA, United States). For each tone, only a stationary plateau part was taken from the original digital recordings of sufficient length. The resulting duration of all auditory stimuli was 100 ms. Artificial rise and fall periods (each 10 ms in duration) were created by linearly decreasing amplitude represented in dB scale in a rising and falling fashion respectively. Mean square amplitudes of all auditory stimuli recordings were digitally equalized. Digital sound editing was done using Anvil Studio (Willow Software, Lake Forest Park, WA, United States), Audacity (Free Software Foundation, Boston, MA, United States), and MATLAB (MathWorks Inc., Natick, MA, United States).

**Table 1:** Response contingencies in the experimental task: this table was read as well as handed in printed form to the participants before the experiment.

	High	Low
Cello	Right button	Left button
Calliope	Left button	Right button

Positive and negative visual feedback stimuli were represented by a black contour thumbs-up sign and thumbs-down sign, respectively. The negative feedback sign was produced by rotating the positive feedback sign by 180°. Stimuli were presented using E-Prime software (Psychology Software Tools, Inc., Sharpsburg, PA, United States) using a high-quality in-ear design stereo headset and a 19″ LSD monitor respectively. The stimuli and the behavioral task were same as used in Novikov et al. (2017).

### Design and Procedure

The auditory two-choice condensation task was used in the experiment (Novikov et al., 2017). The experiment involved six experimental blocks; after the end of each block, participants were offered a short rest.

**FIGURE 1:**
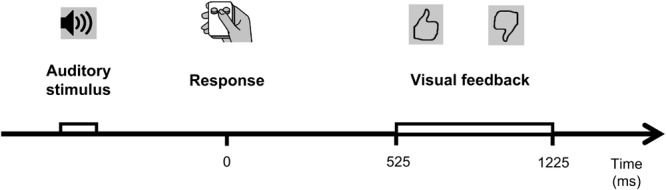
A schematic illustration of the experimental behavioral task. See text for details.

Four stimuli were presented with equal probabilities (25:25:25:25) interleaved in a quasi-random order, 100 stimuli in total in each block. Stimulus onset asynchrony (SOA) ranged from 3500 to 4500 ms (uniform distribution). Participants responded by pressing one of the two specified buttons on a small gamepad with their thumb, using their right hand. A schematic representation of a trial is represented in the Figure 1. The S-R stimulus features (timbre: “cello”/“calliope” and pitch: “high”/“low”) comprising the set of (S-R) mapping table (Table 1) specifies the conjunction contingencies between the two the four stimuli, and the response required to the left and right buttons of the gamepad. Successful execution of the condensation tasks requires mental conjunction of two features, rather than processing any single feature alone (Posner, 1964; Gottwald and Garner, 1975).

Visual feedback was presented 525 ms after the participant’s response for 700 ms. Positive feedback was presented after correct responses, negative feedback was presented after erroneous responses (Figure 1). Feedback was presented only after responses with RTs longer than 300 ms. If RT exceeded 1700 ms, feedback stimuli were additionally supplemented with a word “Faster” on the monitor screen.

Before the experiment, participants were trained to discriminate the four stimuli and remember task rules; during training, they were given a printed table similar to Table 1, which was removed from the participant before the start of experimental blocks. The first experimental block, during which participants achieved stable performance (Lazarev et al., 2014), was not included into all of the following analyses: its purpose was to achieve stable performance. Thus, only the data from the remaining five experimental blocks are reported here (500 trials).

For all of the following analyses, we used only trials with RTs within 300 – 1700 ms range, thus excluding rare cases of exceptionally early responses, as well as abnormally slow responses with a modified “urging” feedback.

### Behavioral Data Analysis

We calculated mean RTs and percentages of correct and erroneous responses, as well as error speeding/slowing score. The latter measure was defined as the mean RT on erroneous trials divided by the mean RT on correct trials. This measure characterizes commission of either fast or slow errors (score below or above 1 correspondingly), in relation to subject’s average correct RTs on the task.

All behavioral data analyses were performed within MATLAB (MathWorks Inc., Natick, MA, United States) using custom-made scripts. Comparisons were made using two-tailed paired *t*-tests.

### Electrophysiological Recording and EEG Preprocessing

The EEG was recorded with an NVX-52 system (Medical Computer Systems, Moscow, Russia) using Neocortex Pro software (Neurobotics, Moscow, Russia) with 27 electrodes in accordance with the modified international 10–10 system and one electrooculogram electrode, with a linked earlobe reference. The band-pass filter was 0.1–200 Hz, and sampling rate was 1000 Hz. The analysis was performed within MATLAB (MathWorks Inc., Natick, MA, United States) using custom-written scripts and built-in functions of EEGLAB toolbox (Delorme and Makeig, 2004). High-amplitude artifacts exceeding 300 μV were rejected from the data. Signals in bad channels were replaced by spherical interpolations over the neighborhood electrodes. Independent component analysis (ICA) was performed, and components related to eye movements were manually selected and rejected from the data. Finally, we substituted signals in channels contaminated with EMG by spherical interpolation over the neighborhood electrodes; we selected for this procedure those channels, in which the spectral power in 25–45 Hz range exceeded 1.5 standard deviations above the mean value taken over the total number of channels × blocks × subjects in the experimental sample (about 2% of channels × blocks × subjects).

In order to reduce effects of volume conduction, current source density transformation (CSD) was performed using open-source CSD toolbox (Kayser and Tenke, 2006).

Response-locked epochs for each condition (correct and erroneous) were extracted from the data (-2000 – 2000 ms relative to the response). Further analysis was conducted only on epochs with the following characteristics: (1) single button press (excluding multiple correcting responses, that could contaminate post-response EEG data), (2) RTs within 300 – 1700 ms range (thus, excluding responses with abnormally short and long RTs), (3) the epoch was preceded and succeeded by correct responses (in order to exclude possible post-error and pre-error effects influencing the trial).

Participants committed less erroneous responses than correct ones, thus, we did trial-matching procedure to equalize variance of mean spectral power estimate between the two conditions. We used all trials from a condition that was less frequent throughout the experiment, and for each of these trials we selected a matching trial from the other condition with the closest RT (each trial was selected only once). In addition to the variance equalization, this procedure equalized mean RTs of the correct and the erroneous trials, thus allowing us to compare these trials on compatible timelines.

### Time-Frequency Analysis: Power

Current source density signal in each channel was translated into time-frequency domain using wavelet transformation with sliding time window. We used Morlet wavelets with frequencies ranging from 2 to 40 Hz in steps of 1 Hz; the numbers of cycles were linearly increasing from 2 (for the lowest frequency) to 37.5 (for the highest frequency), thus providing an equal tradeoff between time and frequency resolutions over the whole frequency range. Centers of the sliding time windows were uniformly distributed over the interval between -1443 and 1442 ms around the response with 20 ms step.

In order to exclude the influence of event-related potentials on the time-frequency data, for each time-frequency bin and each electrode, we calculated non-phase-locked spectral power averaged over subsets of trials that belong to each condition (correct responses and errors). First, we calculated the mean total power by averaging squared norms of complex amplitudes over the trials. Next, we calculated the mean phase-locked power by averaging complex-valued amplitudes over the trials, and then taking squared norm of this sum. Non-phase-locked power was calculated as the difference between the total power and the phase-locked power. We averaged the resulting data within each of the consecutive five time points using a rectangular time window, thus increasing the step of spectral data representation from 20 to 100 ms; this was done in order to improve signal-to-noise ratio.

We performed baseline normalization of the non-phase-locked spectral power, thus obtaining event-related spectral perturbation (ERSP). In order to calculate the baseline, we used stimulus-locked epochs and averaged the spectral power over the time bins with centers in -500 – 0 ms pre-stimulus time window (independently for each electrode and each frequency). After that, we averaged the resulting pre-stimulus powers over the two conditions (correct and erroneous) and used the resulting values as a common baseline.

For the analysis of EEG power, we used two types of paired statistical comparisons: in the first one, we analyzed event-related spectral perturbations (ERSP) for each condition in relation to baseline; in the second one, we analyzed differences in spectral power between conditions (erroneous minus correct). In both cases, spectral power data were converted to decimal logarithms, so both ERSP and cross-condition differences were represented in decibels. Values of non-phase-locked power for each subject and each condition were organized into 4D matrix with the following dimensions: rostrality (7 levels) × laterality (5 levels) × oscillation frequency (12 levels, from 2 to 30 Hz) × time (20 levels, windows centered on time points within the -500 – 1400 ms time interval). For each analysis (ERSP of correct responses, ERSP of errors, and difference between errors and correct responses), we took each data bin separately, and compared the vector, containing individual values in this bin, with zero, using paired *t*-test. For within-condition analysis, this was equivalent to comparing each bin with the baseline; for cross-condition analysis, this was equivalent to comparing the corresponding bins between two conditions. As a result, we acquired a 4-dimensional matrix of *t*-scores for each analysis.

Next, we applied the threshold-free cluster enhancement (TFCE) algorithm (Smith and Nichols, 2009) to this matrix, which resulted in the matrix of TFCE-scores of the same dimensionality. Positive and negative *t*-scores were transformed to TFCE scores using two independent runs of the algorithm. After that, we shuffled the initial data by flipping the sign of all bins in the time-frequency data for randomly selected subset of subjects, and repeated the calculation of TFCE matrix on this shuffled data; this permutational procedure was repeated 1000 times. At each permutation step, we obtained the maximal (positive) and the minimal (negative) TFCE-score over the entire matrix, and then we constructed two distributions: one for the maximal and the other for the minimal values. Finally, for each bin of the non-shuffled TFCE matrix (independently), we calculated the quantiles of “minimal” and “maximal” distributions the value in this bin falls into, thus obtaining permutation-based *p*-value for this bin. Results reported here were considered significant at *p* < 0.05.

For illustrative purposes, we plotted time-courses of power values averaged over alpha-band frequencies (8–13 Hz) and theta-band frequencies (4 – 7 Hz) at selected electrodes.

Also, for illustrative purposes, we plotted scalp maps of the averaged power values over the alpha-band frequencies (8–13 Hz) and over the time points within the pre-response time window (-200 – 0 ms relative to response), as well as over the theta-band frequencies (4 – 7 Hz) and over the time points within the time-window centered on the maximum of feedback-related theta power increase (800 – 1000 ms relative to response, i.e., 275–475 relative to feedback onset).

### Time-Frequency Analysis: Functional Connectivity

We estimated phase synchronization between the midfrontal region and other electrodes. As a measure of synchronization, we used weighted phase-locking index (wPLI) which is an estimation of stability of phase difference between two signals at a given frequency. In contrast to coherence, wPLI is not affected by amplitude modulations and reduces the effects of volume conduction (Vinck et al., 2011).

We calculated wPLI between pairs of signals as suggested by Vinck et al. (2011). First, we calculated coherence between the signals according to the following formula:

$$\begin{array}{c} C(X,Y)=\frac{<XY^{*}>}{\sqrt{<X^{2}><Y^{2}>}} \end{array}$$

where X and Y denotes the two signals, < > – mean, ^∗^ – complex conjugate. Then we extracted wPLI using the formula:

$$\begin{array}{c} wPLI=\frac{\left|<Im(C(X,Y))>\right|}{<|Im(C(X,Y))|>} \end{array}$$

where Im ( ) denotes imaginary part of the complex number, and || – absolute value of the complex number.

For the wPLI analysis, we selected a “seed” midfrontal electrode group – pooled electrodes Fz and Fcz. These two electrodes were selected on the grounds provided by our previous analyses (Novikov et al., 2015, 2017), which were also confirmed within the current time-frequency analysis (see the Results section). These electrodes (especially Fcz) are believed to pick up the signal from the cortical areas comprising the midfrontal hub that implements cognitive control, including performance monitoring and initiation of adjustments following errors (Womelsdorf et al., 2010; Cavanagh and Frank, 2014). In order to pool the two electrodes, we averaged their instantaneous complex amplitude values obtained from the time-frequency analysis, separately for each frequency and for each time point. For each time-frequency bin, we calculated wPLI between a “seed” – midfrontal electrode group (pooled Fz and Fcz) – and each electrode separately. Finally, we averaged the wPLI values over theta-band frequencies (4 – 7 Hz) and over time points within the time-window centered on the maximum of feedback-related theta power increase (800 – 1000 ms). This time window was based on our previous reports (Novikov et al., 2015, 2017), which were confirmed within the current time-frequency analysis (see Results).

Statistical analysis for the wPLI data was performed in the same way as for the time-frequency power data (see above), with the difference that the wPLI data for each subject was organized into 2D (rostrality × laterality), instead of 4D matrix, as this data was averaged over the theta band and the feedback time window. Each bin of the 2D matrix contained the value of wPLI between the electrode corresponding to this bin and the midfrontal electrode group (Fz and Fcz) used as the “seed.” As a result, we obtained baseline-normalized wPLI topography for each condition (correct responses and errors) and wPLI topography for difference between conditions, as well as the corresponding p-values obtained from the TFCE-based permutational statistical analysis.

### Correlation Analysis

In order to test whether negative-feedback-related phase-coupling in the theta band between midfrontal and lateral parietal regions was related to prevalent type of errors a subject committed, we calculated group-level correlation between the cross-condition wPLI difference (erroneous minus correct responses) and the error speeding/slowing score.

For this analysis, we selected the wPLI data of the midfrontal electrode group (pooled Fz and Fcz) vs. P3, P4, CP3, and CP4 electrodes. These pairs were chosen based on the wPLI data obtained (see Results).

For each subject, we took the cross-condition wPLI difference [averaged over the theta-band frequencies (4 – 7 Hz) and over the time points of the feedback time window (800 – 1000 ms relative to response)]. These values across the group of subjects were used to calculate Spearman rank correlation between cross-condition wPLI difference and error speeding/slowing score. The resulting p-values were corrected for multiple electrodes (P3, P4, CP3, CP4) using Bonferroni correction.

## Results

### Behavioral Data

Eight participants were excluded from the analysis due to unsatisfactory behavioral performance or insufficient number of artifact-free erroneous trials. Thus, the results reported here include 43 participants.

Participants made on average 82.1 ± 12.8% of correct responses (mean ± SD) and 17 ± 12.5% of errors. Average RT was 967.3 ± 111.3 ms on correct trials and 1020.3 ± 160.0 ms on errors. RTs for erroneous trials were significantly longer compared with correct trials [*t*(42) = -3.1, *p* = 0.004]. Average error speeding/slowing score was 1.06 ± 0.12, and it was significantly greater than 1 [*t*(42) = 3.02, *p* < 0.01].

### Alpha, Theta, and Beta Spectral Power

**FIGURE 2:**
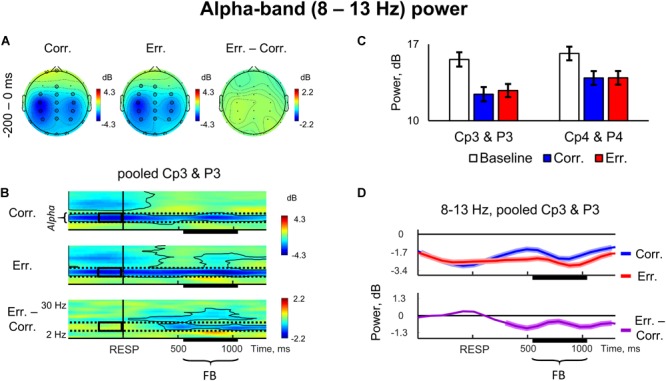
Non-phase locked alpha-band power. **(A)** Topographical maps of baseline-corrected alpha band activity at -200 – 0 ms relative to response (left map represents correct responses, middle map – errors, and right map – difference: errors minus correct responses). Significant electrodes highlighted with black circles (*p* < 0.05, TFCE, permutation statistics). **(B)** Time-frequency plots of baseline-corrected averaged event-related spectral perturbation (ERSP) data at pooled Cp3 and P3 electrodes (top plot – correct responses, middle plot – errors, and bottom plots – difference: errors minus correct responses). Black outlines indicate *p* < 0.05 (TFCE, permutation statistics). Horizontal dashed lines over ERSP plots indicate alpha frequency range (8–13 Hz). Black rectangles represent region of interest: alpha frequency range, -200 – 0 ms pre-response time-window. Time is shown relative to the behavioral response. **(C)** Bar plots representing averaged ERSP data at pooled Cp3 and P3, and Cp4 and P4 electrodes, -200 – 0 ms pre-response time-window. Data are plotted as mean ± standard error of mean. **(D)** Timecourses of baseline-corrected alpha activity averaged at Cp3 and P3 electrodes. Top subpanel: correct and error trials. Bottom subpanel: difference between erroneous and correct trials. Colored contours superimposed on timecourse lines represent statistical significance (*p* < 0.05, TFCE, permutational statistics). Time is shown relative to the behavioral response. “RESP,” behavioral response, “FB,” feedback.

We observed a significant suppression of non-phase-locked alpha-band power (8–13 Hz) on the ERSP for both correct and erroneous trials (Figure 2A–C); the effect was long lasting and included the pre-response time interval (Figure 2B,D) (*p* < 0.05, 4D TFCE-based permutational statistics). In the pre-response time interval, the alpha suppression was most pronounced at the lateral parietal and central electrodes (C3, CP3, P3, C4, CP4, P4) (Figure 2A).

As can be seen in the Figure 2A, the effect was lateralized, with stronger suppression over the left hemisphere. Repeated measures ANOVA on baseline-corrected alpha-band power values over the four posterior parietal electrodes P3, P4, CP3, and CP4 confirmed that laterality factor (left vs. right) was significant [*F*_(1,42)_ = 7.57, *p* = 0.009].

**FIGURE 3:**
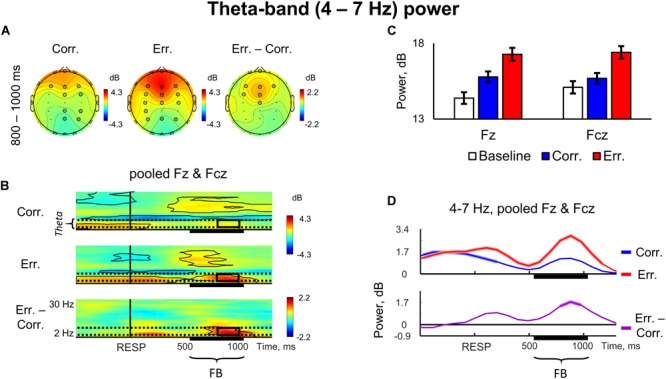
Non-phase locked theta-band power. **(A)** Topographical maps of baseline-corrected theta band activity at 800 – 1000 ms relative to response (left map represents correct responses, middle map – errors, and right map – difference: errors minus correct responses). Significant electrodes are highlighted with black circles (*p* < 0.05, TFCE, permutation statistics). **(B)** Time-frequency plots of baseline-corrected averaged event-related spectral perturbation (ERSP) data at pooled electrodes Fz and Fcz (top plot – correct responses, middle plot – errors, and bottom plot – difference: errors minus correct responses). Black outlines indicate *p* < 0.05 (TFCE, permutation statistics). Horizontal dashed lines over ERSP plots indicate theta (4–7 Hz) frequency range. Black rectangles represent region of interest: theta frequency range, 800 – 1000 ms post-response time-window. Time is shown relative to the behavioral response. **(C)** Bar plots representing averaged ERSP data at Fz and Fcz electrodes, 800 – 1000 ms post-response time-window. Data are plotted as mean ± standard error of mean. **(D)** Timecourses of baseline-corrected theta activity averaged at pooled electrodes Fz and Fcz. Top subpanel: correct and error trials. Bottom subpanel: difference between erroneous and correct trials. Colored contours superimposed on timecourse lines represent statistical significance (*p* < 0.05, TFCE, permutational statistics). Time is shown relative to the behavioral response. “RESP,” behavioral response; “FB,” feedback.

Frontal midline theta power (4–7 Hz) was significantly increased after negative feedback presentation, relative to pre-stimulus baseline (*p* < 0.05, 4D TFCE-based permutational statistics; Figure 3A–C). This effect started 200 ms after the feedback onset and lasted for approximately 500 ms, being maximal within 800–1000 ms after response onset (Figure 3B,D). As it can be seen in Figure 3A, the difference between the two conditions (erroneous trials minus correct trials), was significant at most frontal midline electrodes, with maximum at Fz and Fcz electrodes (*p* < 0.05, 4D TFCE-based permutational statistics).

There were also notable effects in the beta range during feedback presentation. First, as can be seen in Figure 2B, in posterior parietal electrodes there was a significant decrease in beta oscillations on erroneous trials (*p* < 0.05, 4D TFCE-based permutational statistics), and corresponding difference between erroneous and correct responses was also significant within a time interval extending approximately at least from 600 to 1000 ms after response onset (Figure 2B) (*p* < 0.05, 4D TFCE-based permutational statistics). In Figure 3B, representing a frontally located electrodes, one can see a complementary effect – significant increase in beta power during both positive and negative feedback (*p* < 0.05, 4D TFCE-based permutational statistics).

**FIGURE 4:**
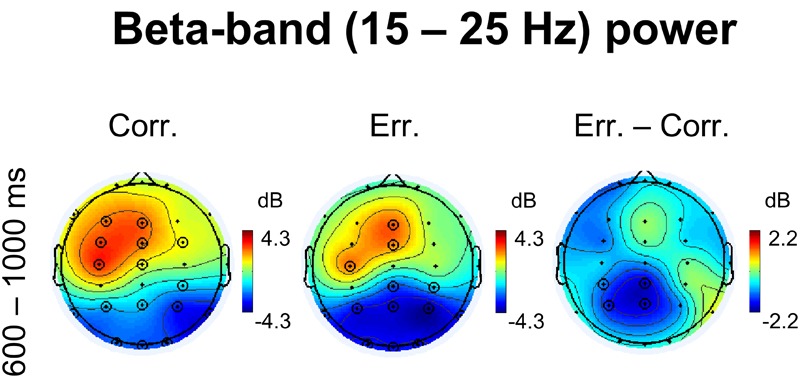
Non-phase locked beta-band power. Topographical maps of baseline-corrected beta-band activity at 600 – 1000 ms relative to response (left map represents correct responses, middle map – errors, and right map – difference: errors minus correct responses). Significant electrodes are highlighted with black circles (*p* < 0.05, TFCE, permutation statistics).

Corresponding topographical maps of beta power within 600–1000 ms after response onset are shown in Figure 4. Indeed, at midline and left frontal electrodes, beta oscillations were significantly enhanced during feedback presentation (*p* < 0.05, 4D TFCE-based permutational statistics). For positive feedback compared with negative feedback, the effect was visibly stronger and involved greater area, yet the difference was not significant (*p* > 0.05, 4D TFCE-based permutational statistics). Posterior decrease of beta oscillations was evidently stronger after negative feedback compared with positive feedback, with the difference between erroneous and correct trials significant at four posterior parietal electrodes – CP3, P3, Cpz, and Pz (*p* > 0.05, 4D TFCE-based permutational statistics). The effect was visibly left-lateralized. Repeated measures ANOVA on baseline-corrected differential beta-band power values over the four posterior parietal electrodes P3, P4, CP3, and CP4 confirmed that laterality factor (left vs. right) was significant [*F*_(1,42)_ = 8.51, *p* = 0.006].

### Theta-Band wPLI Following Feedback

**FIGURE 5:**
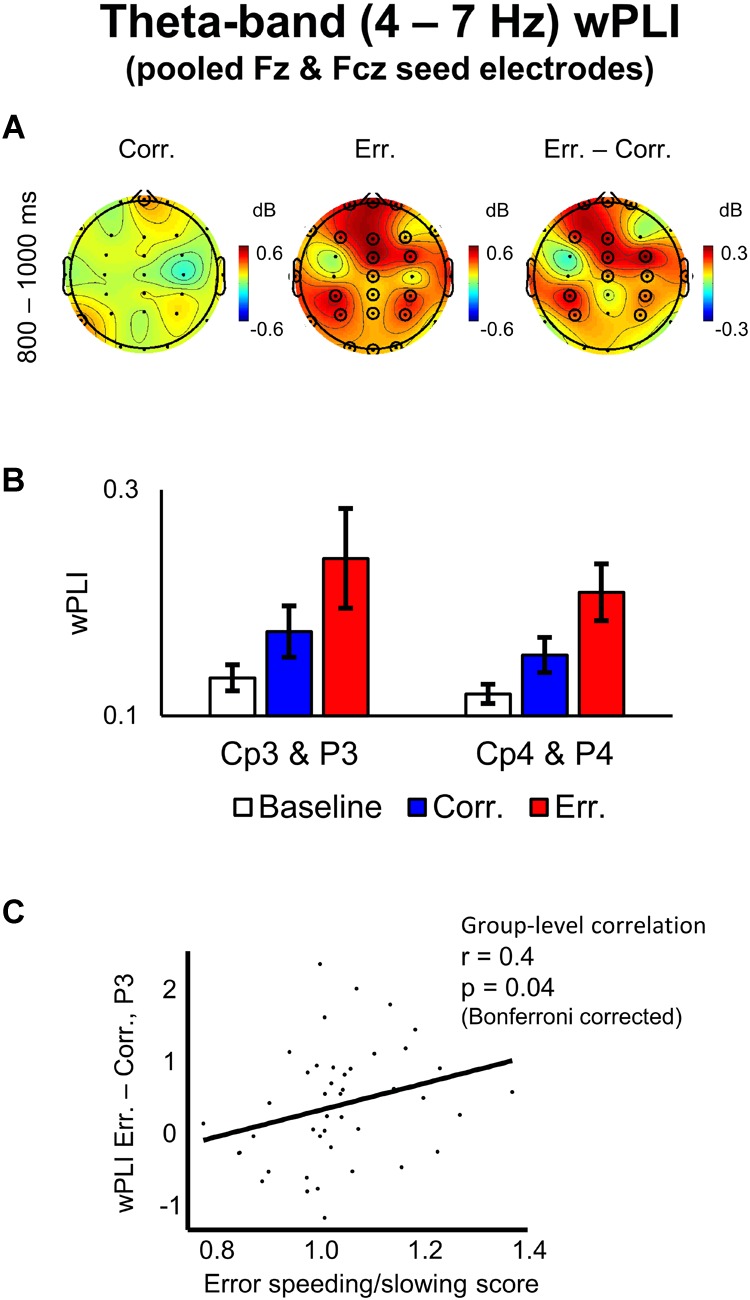
Theta-band wPLI values with pooled Fz and Fcz seed electrodes and its correlation with error speeding/slowing score. **(A)** Topographical maps of baseline-corrected theta-band wPLI values with a seed of pooled electrodes Fz and Fcz at 800–1000 ms relative to response (left map represents correct responses, middle map – errors, and right map – difference: errors minus correct responses). Significant electrodes are highlighted with black circles (*p* < 0.05, TFCE, permutation statistics). **(B)** Bar plots representing wPLI data at left posterior parietal electrodes (pooled Cp3 and P3) and at right posterior parietal electrodes (pooled Cp4 and P4), 800–1000 ms post-response time-window. Data are plotted as mean ± standard error of mean. **(C)** Differential theta-band wPLI values at P3 electrode vs. error speeding/slowing score (RT on erroneous trials divided by RT on correct trials). A regression line is superimposed.

Negative feedback, compared to positive one, was associated with significantly higher wPLI scores (averaged over the theta band frequencies and over the 800–1000 ms post-response time interval) between the midfrontal electrode group (pooled Fz and Fcz) and the following groups of electrodes: (1) frontal electrodes adjacent to the seed electrode group, (2) left parietal electrodes: CP3 and P3, (3) right parietal electrodes: CP4 and P4 (*p* < 0.05, 2D TFCE-based permutational statistics; see Figure 5A,B). The topography of the left and the right parietal groups of electrodes was similar to the topography of the pre-response alpha-band suppression (see above), with the effect, again, being more robust over the left hemisphere – contralateral to the hand used to make responses.

### Correlation Analysis

We conducted a group-level Spearman rank correlation analysis of individual error speeding/slowing score vs. differential wPLI values (errors minus correct trials) between the midfrontal electrode group (pooled Fz and Fcz) and the lateral parietal electrodes CP3, P3, CP4, and P4, each taken separately (wPLI differential values were averaged within the theta frequency band and within the 800–1000 ms post-response time interval). Only for the differential wPLI between the midfrontal electrode group and the P3 electrode, the correlation was significant (*r* = 0.4, *p* = 0.04, Bonferroni corrected; Figure 5C). For other lateral parietal electrodes, no significant correlation was found: CP3 (*r* = -0.03, *p* > 0.05), CP4 (*r* = 0.1, *p* > 0.05), and P4 *(r* = 0.15, *p* > 0.05).

## Discussion

### Overview

In the current study, we used the auditory version of the condensation task to investigate interaction between the cognitive control monitoring system and the decision-making system that occurs after negative feedback presentation on erroneous trials. We analyzed suppression of the oscillatory power in the alpha band preceding a response, FMT power increase after feedback presentation, as well as theta-band coherence between medial frontal and lateral parietal electrodes following negative feedback, and its relationship with RTs on erroneous trials.

### Behavioral Data

Response times (reaching up to 1 s and more) and accuracy (around 82% on average) are typical for the auditory condensation tasks, reported earlier (Lazarev et al., 2014; Novikov et al., 2015, 2017). Note, that the condensation task involves high cognitive load due to a rather complex S-R mapping (Posner, 1964), thus leading to rather long average RTs.

Error RTs where on average significantly greater than correct RTs, thus producing error speeding/slowing score significantly greater than 1. This finding replicates previous reports showing that, during the condensation task, average RTs of erroneous responses are significantly longer than average RTs of correct responses – although the distributions of correct and erroneous RTs significantly overlap (Lazarev et al., 2014; Novikov et al., 2015, 2017). Unlike many tasks commonly used (such as Stroop or flanker task), the stimuli used in this task cannot be classified as “congruent” or “incongruent,” and S-R mapping does not predispose to any overtly predominant responses that tend to be committed prematurely in an “automated” fashion with very small RTs. This feature of the condensation task predisposes to error slowing. Generally, this is typical for attentional tasks, as attentional lapses are known to prolong RTs (Ridderinkhof et al., 2004; Weissman et al., 2006; van Driel et al., 2012). In our task, RT’s are presumably increased even more due to the need for recall and application of the complex S-R mapping rule.

### Alpha-Band Power

We observed depression of the alpha-band power, which was mostly pronounced at lateral parietal and lateral central electrodes during the pre-response time interval. The effect was stronger over the left hemisphere. A very similar pattern of alpha suppression was observed in the study by Cohen and Ridderinkhof (2013), as well as in our previous study, which involved a different version the auditory condensation task (Novikov et al., 2015).

The topography of the lateral posterior parietal regions in which we observed alpha suppression corresponds to an area known as an important hub of decision making. Notable parietal regions presumably involved in decision making are located in the intraparietal sulcus, including the lateral and medial intraparietal areas (LIP, MIP), which are related to sensorimotor transformation during sensory-guided saccade and reach movement planning, respectively (Andersen and Cui, 2009; Cui, 2014; de Lafuente et al., 2015). Adjacent posterior parietal areasV6A and PEc are also implicated in sensorimotor transformations (Breveglieri et al., 2014; Bosco et al., 2016; Hadjidimitrakis et al., 2017; Piserchia et al., 2017). Single-neuron recordings and stimulation studies in animals demonstrated that these areas are clearly involved in evidence integration process in tasks involving spatial decision making (Shadlen and Newsome, 2001; Roitman and Shadlen, 2002; Huk and Shadlen, 2005; Oliveira et al., 2010; Rorie et al., 2010; de Lafuente et al., 2015). Thus, firing rates of a subset of such neurons progressively ramp up, until certain threshold is reached, after which a response is committed (Roitman and Shadlen, 2002). High-level motor planning, action selection and other aspects of decision making are also robustly represented by neuronal discharges within the posterior parietal cortex (Andersen and Cui, 2009; Cui, 2014).

The topography of the effect hints that in addition to posterior parietal cortices, more anteriorly located sensorimotor and premotor areas may be involved. This may be explained by the existence of tight bidirectional anatomical and functional coupling between these areas, and their joint participation in motor planning (Luppino and Rizzolatti, 2000). Apparently, decision making involves action selection and motor planning (which itself requires tight cooperation between premotor, supplementary motor and posterior parietal areas). The motor cortex is also involved in such sensorimotor transformation (Pineda, 2005). Lateralization of the effect in the hemisphere contralateral to the hand used further hints at the involvement of the processes of motor planning.

The effect considered here was rather prolonged – is started well before the response onset and lasted through the response execution. Decision making may be viewed as a continuous process that involves a number of stages, and it apparently starts with evidence accumulation, which has been attributed to posterior parietal areas in numerous studies (Shadlen and Newsome, 2001; Roitman and Shadlen, 2002; Huk and Shadlen, 2005; Oliveira et al., 2010; Rorie et al., 2010; de Lafuente et al., 2015). Application of S-R mapping may be considered a pivotal aspect of decision making, at least in conditions like those used in the current study.

Activation of cortical regions is usually accompanied by alpha-band suppression (Klimesch et al., 2007). This fact could link our findings concerning alpha suppression in lateral parietal areas with the aforementioned single-unit empirical evidence that the parietal cortex is involved in decision making process. This link is additionally supported by the fact that subjects in our task had to select one of the two buttons on each trial, so the task involved sensory-guided motor planning (which is associated with parietal activation, as it was mentioned above). A more robust effect over the left hemisphere could be explained by the fact that subjects committed responses with their right hand.

To the best of our knowledge, there are no experimental studies that directly considered lateralized parietal alpha suppression in EEG data as a correlate of decision making. However, there are MEG studies that demonstrate lateralized posterior alpha suppression, which is mainly localized in the intraparietal sulcus and associated with sensory evidence integration (Van Der Werf et al., 2008, 2010).

In summary, according to the literature data and our results, the lateralized parietal alpha power suppression might partially reflect task-specific processes constituting decision making, which can be viewed as a sensorimotor transformation that converts an integrated sensory evidence into a motor program and involves application of S-R mapping rules. The data obtained do not allow making any straightforward dissociation between the process of action choice and motor-related processes. We believe that such a dissociation might be infeasible due to tight interrelation between multiple aspects of decision making – such as evidence accumulation, action selection, motor planning, etc. – with posterior parietal cortex being an important hub involved in all of these aspects of decision making (Andersen and Cui, 2009; Cui, 2014). In the same way, it seems to be impossible to separate sensory attention and intention to move (Rorie et al., 2010).

### Beta-Band Power

We observed feedback-related enhancement of beta power over the frontal electrodes, with the predominance of the effect on the left side. This effect was apparently stronger for positive feedback, although the difference was not significant. This observation generally replicates our previous finding (Novikov et al., 2017), although in the previous publication the effect was present only for slow responses, while in the current study we analyzed all responses without distinguishing slow and fast ones. This may explain why the effect was reduced in the current study compared to the previous study.

Topography of the effect was similar to that reported previously (Cunillera et al., 2012). Our results generally stay in line with a number of other reports that found enhanced beta-band oscillations in the prefrontal cortex induced by positive feedback during reinforcement learning (Cohen et al., 2007; van de Vijver et al., 2011) and gambling tasks (Marco-Pallares et al., 2008). Generally, beta oscillations are believed to reflect involvement of frontal, striatal, and hippocampal structures related to memory during reward processing (Mas-Herrero et al., 2015).

We also observed differential suppression of beta oscillations during negative feedback in the posterior parietal areas. Beta enhancement may be interpreted as preservation of the status quo (Engel and Fries, 2010), while suppression of beta oscillations may be described as a dynamic state, that includes memory update (Novikov and Gutkin, 2018). Importantly, the differential effect was statistically significant specifically at the posterior parietal electrodes, including Cp3 and P3 electrodes, thus confirming the role of the posterior parietal area in the S-R mapping.

### Theta-Band Power

Frontal midline theta power was significantly higher after the negative feedback compared to the positive one. This finding replicates numerous studies (Cohen et al., 2007; Marco-Pallares et al., 2008; De Pascalis et al., 2012; Li et al., 2016; Novikov et al., 2017). Such FMT increase reflects external error detection, signaling the need for increase in cognitive control, and it is considered an event that initiates a sequence of neural adjustments in task-related systems (Cavanagh et al., 2011; Cohen and van Gaal, 2013; Mas-Herrero and Marco-Pallares, 2014). It is also known that FMT power increase associated with negative feedback is involved in subsequent behavioral adjustments and task learning. For example, the feedback-related FMT is larger on erroneous trials followed by correct ones, compared to erroneous trials followed by another error (van de Vijver et al., 2011).

### Theta-Band Functional Connectivity

In the current study, functional coupling (assessed as wPLI score) between medial frontal and lateral parietal regions in the theta band was significantly higher after the negative feedback presentation compared to the positive one. This finding presumably suggests that negative feedback induces enhanced interplay between the midfrontal performance monitoring system and the parietal associative systems involved in decision making (including sensory evidence integration and reactivation of S-R mapping). This finding is in agreement with the idea that theta-band coupling reflects top–down modulations of distant areas aimed at producing neural and behavioral adjustments (Varela et al., 2001).

Previous studies reported theta-band coupling of the midfrontal region with the dorsolateral prefrontal cortex (Cavanagh and Frank, 2014; Luft et al., 2014), left-central motor areas (van de Vijver et al., 2011), posterior parieto-occipital visual regions (Cohen and van Gaal, 2013), as well as with subcortical structures such as the subthalamic nucleus, which is critical for motor inhibition (Zavala et al., 2014). Correlations between theta-band oscillations in the frontal areas and beta-band oscillations in the posterior areas (including both occipital and posterior parietal cortices) were also recently reported (van de Vijver et al., 2018). These various interactions are likely to reflect different aspects of adjustments in situations when cognitive control should be increased. In contrast to those studies, in our study we observed theta-band coupling between midfrontal and lateral parietal regions – specifically the electrodes CP3, P3, CP4, and P4. Note, that only the midfrontal region was selected *a priori* as the seed for the functional connectivity analysis, so our finding that this region is most strongly connected with the lateral parietal sites was purely data-driven. Within posterior areas, the differential effect did not involve occipital electrodes and was maximal at CP3 and P3 electrodes; thus, in contrast to a number of studies that demonstrated parieto-occipital effects (Cohen and van Gaal, 2013; van de Vijver et al., 2018), the current study evidences involvement of the posterior parietal areas rather than of the low-level sensory visual areas. Additionally, we observed phase coupling of midfrontal electrodes with more lateral frontal electrodes, thus replicating previous findings of theta-band synchronization between the midfrontal region with the dorsolateral prefrontal cortex (Cavanagh and Frank, 2014; Luft et al., 2014).

The specific pattern of functional connectivity observed could be explained by the fact that the condensation task we used implies rather non-straightforward decision-making process with complex S-R mapping rule based on feature conjunction. Consequently, a large proportion of errors during this task presumably arises from failures of S-R mapping rule reactivation. Assuming that the lateral posterior parietal regions are critically involved in the processes related to decision making, it is likely that post-error neural adjustments would mainly occur in these regions. According to this logic, the functional coupling we observed after erroneous responses is a hallmark of top-down influence from the midfrontal performance monitoring system to lateral parietal decision-making systems, aimed to provide neural adjustments for improving future task performance. These adjustments may, theoretically, include: (1) optimization of stimulus processing by raising excitability of neural populations selective for the stimuli features used in the task, (2) inhibiting the populations selective for other auditory features or for information from other sensory modalities, (3) raising excitability or stabilizing background activity of neural populations that implement correct S-R mappings, (4) inhibiting populations that implement incorrect S-R mappings.

### Correlation of Theta-Band With Error Slowing

We calculated for each subject a behavioral measure “error speeding/slowing score,” which was defined as the ratio between erroneous and correct RTs. This measure served as an estimate of subject’s predisposition to commit either fast or slow errors, irrespective to his or her average speed on the task. Then we correlated error slowing with cross-condition difference (errors minus correct trials) of wPLI between midfrontal and lateral parietal sites in the time interval after feedback onset. The results suggested that participants, who tended to commit slower errors, demonstrated stronger negative-feedback-induced functional coupling between the midfrontal and the left parietal region.

It was previously reported that feedback-related FMT signal is more pronounced on those trials, on which feedback is more useful and informative (Cohen et al., 2007). In our previous study (Novikov et al., 2017), we demonstrated that errors with longer RTs are associated with the state of high response uncertainty and followed by the state of high outcome uncertainty.

We understand uncertainty here as a broader concept than a conflict between activated motor programs, and we presume that uncertainty may embrace a continuum of brain states including the one with no motor programs sufficiently activated. This may happen as a result of a failure of task-specific processes related to decision making – such as recognition of a stimulus and application of a proper S-R mapping rule. Such failures may be caused by attentional lapses (Weissman et al., 2006).

In conditions of high outcome uncertainty, feedback is the only source of information about accuracy of the response committed. In the present study, we hypothesized that the negative-feedback-induced functional interaction between the midfrontal sites and regions involved in decision making (which presumably links error detection with the following task-specific neural adjustments) should be also more pronounced in the situation of highly informative feedback, i.e., after slow errors. Thus, the results we obtained from the correlation analysis confirmed our hypothesis. A complementary explanation may be that since slow errors were likely caused by failures within task-specific systems of decision making rather than by failures of non-specific motor inhibition, the top–down modulation of the regions related to decision making was more pronounced after the slower errors compared with the faster ones.

The correlational findings reported here supposedly reflect differences in the main type of errors committed by participants when performing the task. Those participants who committed slower errors, experienced greater response uncertainty preceding errors, which delayed response commission. This state was supposedly caused by failures of task-specific processes, and for such participants outcome uncertainty was also enhanced, making feedback more important to them after such slow errors. Those participants, who committed faster errors, presumably, committed relatively more errors due to lowered motor threshold rather than due to compromised task-specific processes of decision making. Thus, they supposedly experienced less uncertainty, and external feedback was less important for them.

## Conclusion

In the present study, we investigated functional interaction between the performance monitoring and the decision-making systems of the brain, using EEG analysis of the data collected in the auditory version of condensation task. This task involves much more complex S-R mapping than most tasks commonly used in the field such as the tasks like Stroop task, Eriksen task, Simon task, or SART (Posner, 1964; Gottwald and Garner, 1975), and thus creates greater load on the representations of S-R mapping and sensorimotor transformation. Specifically, we were interested in the interaction that occurs after negative feedback presentation, which followed erroneous responses. First, we demonstrated that certain stages of decision making process involve the posterior parietal regions, as reflected by alpha-band suppression in the corresponding electrodes in the time window between stimulus and response. Additionally, we demonstrated that the posterior parietal regions are also involved in adjusting S-R mapping following errors as evidenced by differential beta-band suppression in response to a negative feedback. Second, we confirmed a well-known effect of midfrontal theta increase after negative feedback presentation. Third, we demonstrated that functional interaction in the theta band between the midfrontal and the lateral posterior parietal region (measured with wPLI) was significantly stronger after the negative feedback compared with the positive one. We interpret this interaction as a hallmark of top–down signal that originates in the midfrontal region (which detects the need for increased cognitive control due to negative feedback reception) and leads to neural adjustments in the decision-making regions.

Finally, we demonstrated that the negative-feedback-related increase of the functional coupling between the aforementioned regions was more pronounced for subjects who tended to commit slower errors. This effect could be explained by the following two facts: (1) slow errors lead to the state of high uncertainty, which makes the feedback highly informative, while fast errors are more likely to be detected internally, and (2) fast errors are presumably caused by motor inhibition failures which are not related to task-specific processes occurring in the considered decision-making regions.

In summary, we demonstrated functional coupling between the regions involved in performance monitoring and decision making, respectively, that occurs after negative feedback presentation and is more pronounced when potential neural adjustments initiated by this interaction are more relevant.

## Ethics Statement

The experiments were carried out in accordance with the Declaration of Helsinki and its amendments and were approved by the Institutional Review Board of the National Research University Higher School of Economics. Each participant signed informed consent before the experiment.

## Author Contributions

YN, NN, NZ, and BC conceived and designed the work. YN and NZ acquired the data. YN, NZ, and NN analyzed the data. YN, NN, NZ, and BC interpreted the data. YN and NZ drafted the work. YN, NN, NZ, and BC revised the work critically for important intellectual content. YN, NN, NZ, and BC approved the final version to be published and agreed to be accountable for all aspects of the work in ensuring that questions related to the accuracy or integrity of any part of the work are appropriately investigated and resolved.

## Conflict of Interest Statement

The authors declare that the research was conducted in the absence of any commercial or financial relationships that could be construed as a potential conflict of interest.
